# Lifelong physical activity is associated with promoter hypomethylation of genes involved in metabolism, myogenesis, contractile properties and oxidative stress resistance in aged human skeletal muscle

**DOI:** 10.1038/s41598-018-37895-8

**Published:** 2019-03-01

**Authors:** M. Reza Sailani, Jens Frey Halling, Henrik Devitt Møller, Hayan Lee, Peter Plomgaard, Henriette Pilegaard, Michael P. Snyder, Birgitte Regenberg

**Affiliations:** 1 Department of Genetics, Stanford University School of Medicine, Stanford, USA; 2 Department of Biology, University of Copenhagen, Copenhagen, Denmark; 3 The Centre of Inflammation and Metabolism, The Centre for Physical Activity Research, Department of Clinical Biochemistry, Rigshospitalet, Copenhagen University Hospital, Copenhagen, Denmark

## Abstract

Lifelong regular physical activity is associated with reduced risk of type 2 diabetes (T2D), maintenance of muscle mass and increased metabolic capacity. However, little is known about epigenetic mechanisms that might contribute to these beneficial effects in aged individuals. We investigated the effect of lifelong physical activity on global DNA methylation patterns in skeletal muscle of healthy aged men, who had either performed regular exercise or remained sedentary their entire lives (average age 62 years). DNA methylation was significantly lower in 714 promoters of the physically active than inactive men while methylation of introns, exons and CpG islands was similar in the two groups. Promoters for genes encoding critical insulin-responsive enzymes in glycogen metabolism, glycolysis and TCA cycle were hypomethylated in active relative to inactive men. Hypomethylation was also found in promoters of myosin light chain, dystrophin, actin polymerization, PAK regulatory genes and oxidative stress response genes. A cluster of genes regulated by GSK3β-TCF7L2 also displayed promoter hypomethylation. Together, our results suggest that lifelong physical activity is associated with DNA methylation patterns that potentially allow for increased insulin sensitivity and a higher expression of genes in energy metabolism, myogenesis, contractile properties and oxidative stress resistance in skeletal muscle of aged individuals.

## Introduction

Regular physical activity promotes health and longevity. The Harvard Alumni Health Study revealed that regular exercise reduces mortality in elderly men^1^ and in the Norwegian male population, half of the people with high physical fitness reach the age of 85 compared to only one fourth among men with low physical fitness^2^. A physically active lifestyle is associated with a number of physiological, biochemical and transcriptional changes contributing to reduced risk of developing type 2 diabetes (T2D) and cardiovascular diseases during aging^3^. Among the beneficial effects of regular exercise is maintenance of muscle mass through enhanced proteostasis of structural proteins such as actin and myosin^4^. In addition, exercise activates several signaling cascades that ultimately initiate transcriptional regulation of genes encoding proteins involved in mitochondrial function and other pathways associated with energy metabolism^5,6^.

However, little is known about epigenetic mechanisms that might contribute to the beneficial effects of regularly performed exercise in aged individuals. Epigenetic modifications are known to constitute an important regulatory layer of transcription and DNA methylation is a major epigenetic modification that governs chromatid structure and gene expression. Methylation of promoter regions is associated with repression of transcription, and DNA methylation will therefore typically cause gene silencing^7^. Although patterns of DNA methylation are mainly established early in development and traditionally have been thought to be largely maintained in the differentiated cells, several environmental factors have been shown to affect DNA methylation^8^. Hence, physical activity has been found to induce changes in DNA methylation patterns as well as expression of corresponding genes in skeletal muscle, both in response to a single exercise bout^9^ and after prolonged exercise training^10,11^. In addition, the cellular aging process seems to be tightly linked with complex epigenetic changes. Aging has been associated with global hypomethylation of the genome^12^ although a recent study in aging humans suggest that DNA in muscle is generally hypermethylated as we age^13^.

The aim of the present study was therefore to investigate the impact of lifelong physical activity on global DNA methylation patterns in muscle tissue of 16 healthy aged men with an average age of 62 years. Eight of these subjects had a physically active lifestyle with regular exercise their entire lives and eight subjects were healthy but with a physically inactive lifestyle. Our data reveal physical activity level dependent epigenetic modifications in genes involved in muscle structural dynamics, energy metabolism, and predisposition to T2D. This suggests that the level of physical activity throughout life affects DNA methylation of genes within cellular pathways and metabolic processes in skeletal muscle.

## Results

To test how lifelong endurance exercise training influences epigenetic imprinting, we measured global DNA methylation in muscle tissue of two groups of healthy 60–65 year old men. One group of 8 men reported high levels of physical activity through regular exercise training their entire lives, while another group of 8 men reported low lifelong levels of physical activity. While height and weight were similar between the two groups, body fat percentage was 39% higher with a corresponding 13% lower percentage of lean mass in the inactive than the active men (Table 1). Moreover, cycling endurance was 41% lower in the inactive group than the active indicating a large difference in overall physical fitness (Table 1).

**Table 1:** Subject characteristics.

Parameter	Physically inactive (n = 8)	Physically active (n = 8)
Age (years)	62.8 ± 1.4	62.1 ± 1.3
Height (cm)	176 ± 8	180 ± 9
Body weight (kg)	82.9 ± 12	82.3 ± 14
Fat %	30.5 ± 4.2	21.9 ± 7.9*
Lean %	66.3 ± 7.9	74.8 ± 4.3*
Cycling test duration (min)	13.1 ± 5.1	20.8 ± 3.6*
Cycling test wattmax	188 ± 51	266 ± 36*
Total blood cholesterol (mmol/L)	5.73 ± 0.88	5.44 ± 1.16
HDL cholesterol (mmol/L)	1.50 ± 0.46	1.60 ± 0.47
LDL cholesterol (mmol/L)	3.54 ± 0.78	3.41 ± 0.81
Free testosterone (ng/dL)	12.40 ± 4.08	12.69 ± 6.36
C-reactive protein (mg/L)	1.71 ± 0.76	1.00 ± 0.76
Total leukocyte count (x10^9^/L)	7.11 ± 1.70	6.81 ± 1.84

Measured physiological parameters of healthy men with a life-long sedentary or life-long physically active life-style (each group n = 8), showing mean ± standard deviation. Cycling endurance and percent body fat separated the two groups significantly (p-values, * < 0.05). Selection of men in the two groups was based on questionnaires, in which the sedentary group of men (n = 8) reported a lifelong sedentary lifestyle with physical activity at most one time per week throughout their life, while the group of physical active men (n = 8) had exercised throughout their life more than three times per week.

### Whole genome methylation pattern

**Figure 1:**
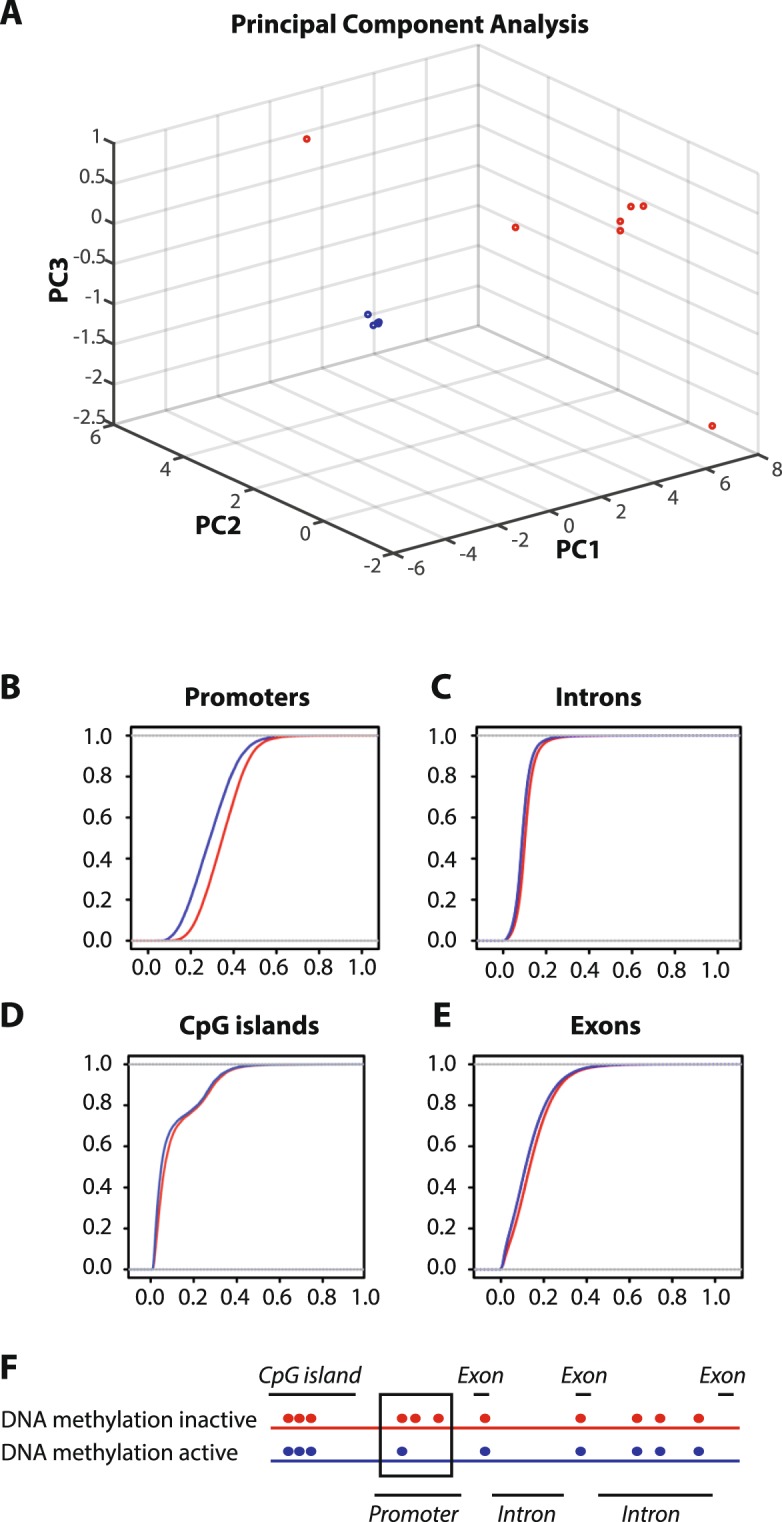
DNA methylation patterns in men with a physically active lifestyle and physically inactive lifestyle. (**A**) PCA analysis based on methylation profile of 26 CpG loci from muscle tissue. The 60–65 year old men with a lifelong physically active lifestyle are presented in blue color (n = 8) and those with a lifelong physically inactive lifestyle are presented in red (n = 8). (**B**–**E**) Cumulative distribution of the average DNA methylation level in active and inactive men for (**B**) promoters, (**C**) introns, (**D**) CpG islands, (**E**) exons in the human genome. (**F**) Model of methylation patterns (represented by dots) in DNA (represented by lines) of men with a physically active and inactive lifestyle.

DNA methylation in muscle tissue was analyzed by whole genome sequencing of bisulfate-converted DNA using Illumina paired–end sequencing. To evaluate the promoter methylome, we first calculated the average methylation in promoters for each of the two groups, defining promoters as −2 kb to +1 kb relative to transcription start sites (TSS) of 28857 genes (Fig. 1B, Supplementary Dataset 1). Principle component analysis (PCA) showed a clear division between methylation profiles of trained and untrained individuals (Fig. 1A). Moreover, methylation levels were generally lower in promoters of the active group than the inactive group (Fig. 1B). Of these, 714 promoters were significantly less methylated in physically active than inactive subjects (Fischer test, FDR < 0.001 with at least 30% reduction in methylation, Supplementary Dataset 2), while a small number of promoters had increased methylation levels in physically active compared with inactive (31 promoters, Supplementary Dataset 3). We also investigated the genome wide DNA methylation levels for introns, CpG islands and exons in the active and inactive men, but found no difference between the two groups (Fig. 1B–E). Thus, at the whole genome level, only promoter regions displayed altered epigenetic profiles in response to lifelong physical activity (Fig. 1B–F).

### Epigenetic regulation of genes involved in key metabolic pathways

**Figure 2:**
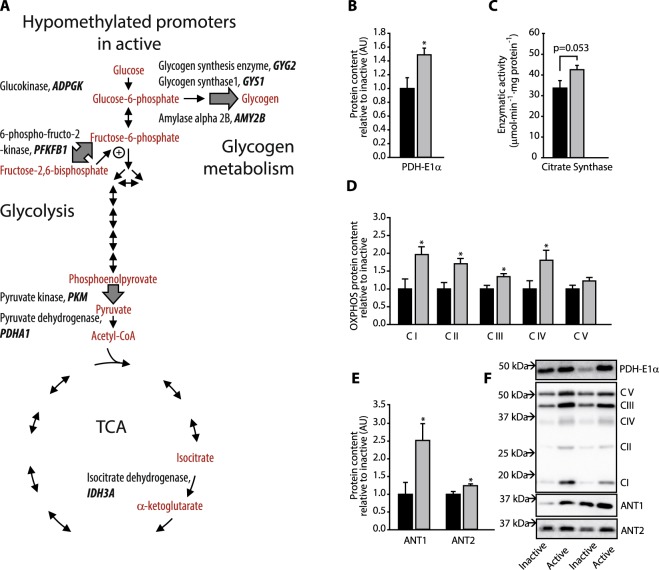
Regulation of key enzymes in the central carbon and energy metabolism at the level of promoter DNA methylation and protein level. (**A**) Genes with significant and >30% hypomethylated promoters are shown in bold. Corresponding substrates and products for the affected steps in glycolysis, glycogen metabolism and TCA cycle are shown in red, insulin sensitive steps are shown as broad grey arrows. Protein levels of (**B**) pyruvate dehydrogenase, (**D**) OXPHOS subunits, (**E**) and mitochondrial ATP-transporters are shown as average of the level in eight physically active men (grey bars) and eight physically inactive men (black bars). *Indicates p < 0.05. (**F**) Representative Western blots for four men. (**C**) Citrate synthase activity measured as enzymatic activity. See Supplementary Figs 1 and 2A–C for full-length western blots and scatter plots showing individual data points for all presented proteins.

Among the 714 genes with significantly less methylated promoters in the active group were several genes encoding enzymes involved in energy metabolism (Fig. 2A). This list included genes encoding key glycogen synthesis enzymes, glycogenin 2 (*GYG2*), glycogen synthase 1 (*GYS1*), as well as the glycogen degrading amylase alpha 2B (*AMY2B*). The result supports a model in which DNA-methylation patterns allow a higher capacity for expression of genes involved in glycogen storage and metabolism in active than inactive men (Fig. 2A, Supplementary Dataset 2). Promoter hypomethylation was also found in key glycolysis and TCA cycle genes. The list included genes encoding ADP dependent glucokinase (*ADPGK*), a functional homologue of hexokinase that catalyzes the phosphorylation of glucose to glucose 6-phosphate using ADP as substrate, and the muscle specific pyruvate kinase (*PKM*), which mediates the last step in glycolysis. Similarly, promoter methylation of the gene encoding 6-phosphofructo-2-kinase/fructose-2,6-biphosphatase (*PFKFB*) was lower in the active relative to inactive subjects, which could potentially increase the capacity for regulation of glycolysis. Also, the promoter regions of genes encoding the pyruvate dehydrogenase alpha 1 subunit (*PDHA1*) that links glycolysis and the TCA cycle, and the rate limiting enzyme in TCA cycle flux, isocitrate dehydrogenase (*IDH3A*), were found to be hypomethylated in the active compared with inactive subjects.

To investigate whether the increased capacity for expression of central glycolytic and TCA cycle genes was also evident at the protein level, we analyzed key enzymes in oxidative metabolism by western blotting and enzyme activity assays. We confirmed that the *PDHA1* product, pyruvate dehydrogenase E1 alpha, was higher at the protein level in active than inactive men (Fig. 2B). Also, the activity of citrate synthase tended to be higher (p = 0.053, in muscle from active than inactive men), supporting an increased capacity for flux through the TCA cycle in muscle tissue of active men (Fig. 2C).

Mitochondrial oxidative capacity sets the limit for aerobic energy metabolism in muscles. The level of subunits in oxidative phosphorylation Complex I, II, III and IV was significantly higher in active men than inactive men (Fig. 2D). Interestingly, six genes in the mitochondrial transporter family SLC25 exhibited marked promoter hypomethylation in the active compared with the inactive group (Supplementary Dataset 2). This included *SLC25A5* that encodes an isoform of the mitochondrial adenine nucleotide translocator (ANT) family, which catalyzes the exchange of ATP produced in the mitochondria with ADP from the cytosol. Western blots revealed that protein content of both ANT1 and ANT2 were indeed higher in active than inactive men (Fig. 2E). Altogether, the present results suggest that in lifelong physically active men, epigenetic regulation increases the basal expression of key rate-limiting steps in glycogen synthesis, glycolysis, TCA cycle, mitochondrial electron transport complexes and ADP/ATP exchange.

### Epigenetic link between mitochondrial regulation and Wnt/GSK3 signaling

**Figure 3:**
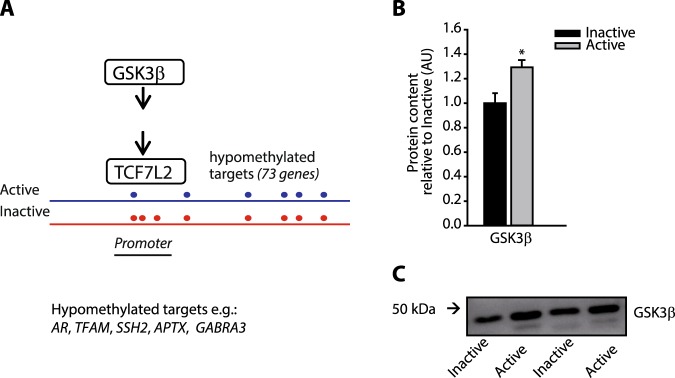
Hypomethylation of 73 GSK3β /TCF7L2 targets. (**A**) Model of 73 promoters known to bind TCF7L2. These promoters were hypomethylated (represented by dots) in men with a physically active lifestyle (blue) compared to men with an inactive lifestyle (red). (**B**) Protein levels of GSK3β are shown as average of the level in eight physically active men (grey bars) and eight physically inactive men (black bars). *Indicates p < 0.05. (**C**) Representative Western blots for four men. See Supplementary Figs 1 and 2D for full-length western blot and scatter plots showing individual data points for GSK3β.

In addition to rate-limiting enzymes in glycolysis and TCA cycle, we found that promoter regions of genes encoding important insulin responsive proteins involved in carbon metabolism were hypomethylated in active compared with inactive men, suggesting that promoter methylation links regulation of metabolic pathways with higher capacity for insulin action in skeletal muscle. To understand how mitochondrial capacity might be regulated at the epigenetic level and integrated with insulin signaling, we searched for overrepresented groups of hypomethylated promoter regions underlying potential mitochondrial regulators. Of particular interest, the promoter regions of 73 genes regulated by the transcription factor 7-like 2 (TCF7L2) were all hypomethylated in the active relative to the inactive men (Fig. 3A and Supplementary Dataset 4). The mitochondrial transcription factor (TFAM), that controls the biogenesis of mitochondria through its role as the central transcription factor of mtDNA^14^, was among these. TCF7L2 is part of the glycogen synthase kinase GSK3/β-Catenin signaling pathway that is stimulated by both Wnt and insulin signaling^15^, and regulates mitochondrial metabolism and insulin sensitivity by transcriptional induction^16,17^. Promoters of *TCF7L2* and *GSK3* were not themselves hypomethylated, however the protein level of GSK3β was significantly increased in the active group relative to the inactive (Fig. 3B).

### Myogenesis and muscle structural dynamics

**Figure 4:**
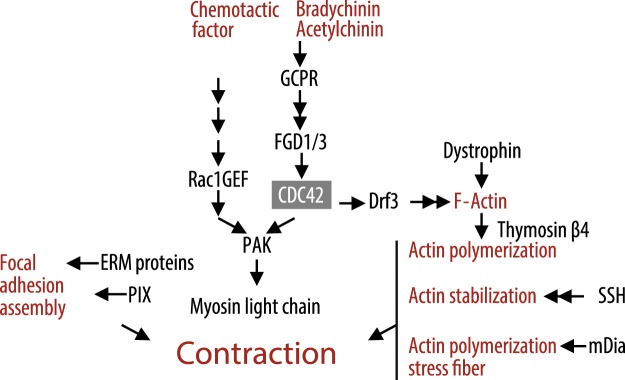
Promoter hypomethylation of genes involved in myogenesis, structural dynamics of muscles and CDC42 signaling. Shown in black are gene products with significant and >30% hypomethylated promoters in the physically active men compared with inactive men. A grey box indicates the pathway position of CDC42. Pathway steps are indicated with black arrows. Pathway end products and inducers are show in red text. The model is based on map 04810, http://www.genome.jp/kegg/pathway.html.

In addition to metabolism, the Wnt signaling pathway is also known to regulate muscle fiber differentiation through MyoD and other myogenic factors^18^. This led us to further analyze promoter methylation of genes involved in myogenesis and muscle structural dynamics. Indeed, the promoter region of the *MYOD1* gene was relatively hypomethylated in the active versus the inactive group (Supplementary Dataset 2). Furthermore, promoters of genes involved in muscle contraction were hypomethylated, suggesting that the transcriptional capacity of genes involved in the skeletal muscle contractile apparatus was higher in active men than in inactive men (Fig. 4). Most importantly, this involved the *MLC* gene encoding Myosin Light Chain, a component of the principal force generating motor protein in skeletal muscle. Promoter hypomethylation was also found in genes encoding Ezrin/Radixin/Moesin (ERM) proteins that crosslink actin filaments with the plasma membrane, mDIA, which participates in formation of actin stress fibers important for establishment of cellular tension, the ADF/cofilin gene, *SSH1*, involved in actin dynamics and the dystrophin gene, *DMD*, which links sarcolemma to the contractile apparatus. A number of genes (*GCPR*, *FGD1/3*, *DRF3*, *PAK*, *PIX*, *Rac1GEF*) involved in the CDC42 signaling pathway, which regulates actin/myosin cross-linking and actin polymerization^19^, also exhibited promoter hypomethylation in the active relative to inactive subjects. In addition, the promoter of the smooth muscle repair protein thymosin-4 peptide, TMSB4, was relatively hypomethylated in active subjects. Overall, the results show that promoters of numerous genes involved in myogenic, contractile, structural and reparation properties of skeletal muscle were hypomethylated in the active compared with inactive men (Fig. 4 and Supplementary Dataset 2).

### Oxidative stress resistance

**Figure 5:**
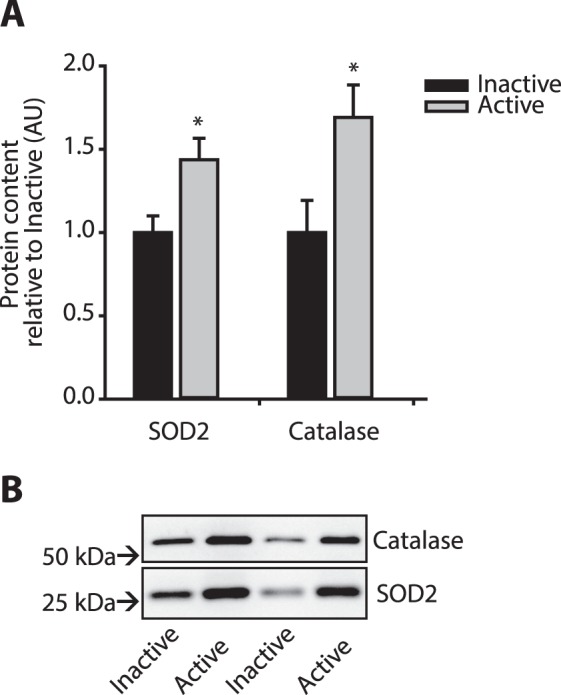
Protein levels of catalase and super oxide dismutase, SOD2, in men with a physically active lifestyle and physically inactive lifestyle. (**A**) Protein levels of catalase and SOD2 are shown as average of the level in eight physically active men (grey bars) and eight physically inactive men (black bars). *Indicates p < 0.05. (**B**) Representative Western blots for four men. See Supplementary Figs 1 and 2E for full-length western blot and scatter plots showing individual data points for SOD2 and catalase.

In general, promoter regions of genes involved in the oxidative stress response was hypomethylated in active men. Microsomal glutathione S-transferase 1 gene, *MGST1*, and the neuroprotective *OXR1* gene had reduced promoter methylation, and the protein level of both catalase and superoxide dismutase 2 (SOD2) were significantly higher in muscle tissue of physically active men than inactive men (Fig. 5) in accordance with the hypomethylation of the corresponding *SOD2* and *CAT* promoters (20% and 18%). To test if the oxidative stress response in trained men rendered muscle tissue more resistant to oxidative stress, we measured protein carbonylation as an indicator of oxidative damage. Carbonylation of proteins was 29% lower in tissue of active men, supporting a higher tolerance to oxidative stress in this group (Result in^20^).

Mitogen activated protein kinases, MAPKs, can be activated by ROS and are directly involved in induction of antioxidant genes^21,22^. A negative regulator of the MAPK p38 pathway, PTPRR, displayed reduced promoter methylation, suggesting a potential for reduced p38 pathway activity in the tissue of active men. Recent studies of muscle stem cells from aged mice revealed that inhibition of the p38 pathway, activated muscle stem cells, which thereby improved the potential for regeneration of damaged muscle^23^.

### Stem cell potential

Because target genes of the stem cell regulating GATA3 and MYC1 were also found to be hypomethylated in active men (Supplementary Dataset 4), we speculated that the satellite stem cell number might be higher in muscle from physically active than inactive men. The PAX7 protein is specific for satellite stem cells in skeletal muscle and has previously been used as marker for stem-cell count in muscle^24^. However, we found no difference in the protein content of PAX7 between trained and untrained muscle (data not shown), suggesting that the number of stem cells was similar in muscle from the two groups of men, although this does not exclude that the stem cell potential is higher in tissue from physically active men than from inactive men.

### Polycomb target genes

Although aging generally leads to hypomethylation of DNA, tumor suppressor genes and polycomb targets have been shown to display hypermethylation with increasing age^12^. This effect appeared to be absent in the physically active group, where promoter regions of 62 polycomb targets were hypomethylated, suggesting that polycomb methylation of Histone H3K27Me3 and subsequent inactivation of polycomb genes was delayed in the physically active group (Supplementary Dataset 4). We measured protein levels of the enzymatic component of the polycomb repressive complex 2, EZH2, but found no differences in the content of EZH2 in muscle from physically active and inactive men (data not shown). Furthermore, we found no correlation between hypomethylated genes in our study and genes known to carry the H3K27Me3 mark in muscle (Supplementary Dataset 5).

### Promoter hypomethylation of a methyltransferase

To understand how differential DNA methylation may arise in the muscle tissue of active compared with inactive men, the list of hyper- and hypomethylated genes for DNA methyltransferases and demethylases was examined. The promoter of the DNA methyltransferase gene *DNMT3B* was hypomethylated (30%) in physically active men relative to inactive men, suggesting increased capacity for transcription of this methyltransferase gene and hence higher de novo methyltransferase capacity in skeletal muscle with lifelong physical activity. However, the levels of DMNT3B protein were similar in the physically active and inactive men, indicating that the capacity for higher *DNMT3B* expression was apparently not evident at the protein level (data not shown).

## Discussion

This study presents a library of changes in global DNA methylation patterns in skeletal muscle that occurs during lifelong physical activity which serves as a resource for future research. Of particular interest, we show that lifelong physical activity was associated with differential promoter DNA methylation levels in skeletal muscle with effects on rate limiting and insulin sensitive steps of glycolysis and TCA cycle as well as muscle regeneration.

It is well established that exercise prevents or delays the loss of muscle mass, insulin sensitivity and the ability to repair muscle damage during aging^3,25,26^, but how this occurs at the epigenetic level in skeletal muscle is unclear. The observed methylation profiles suggest that the capacity for glycolytic as well as TCA cycle flux is higher in muscle from physically active than inactive men through promoter hypomethylation of several key genes in the active subjects (Fig. 2A). The promoter methylation profiles also suggest that epigenetic modulation contributes to improved insulin action in the physically active men, because the modulated glycolytic genes were particularly encoding important insulin sensitive steps of glycolysis (*PFKFB1*, *PKM* and *GYS1*).

A six month exercise invention was reported to increase the absolute level of methylation in DNA from adipose tissue of younger men^27^. This study identified 600 promoters that were hypermethylated in response to exercise training. While these hypermethylations are in contrast to the general observation in the current study, there is a general trend in both studies for altered promoter methylation of genes involved in T2D via the insulin- and Wnt-sensitive transcription factor TCF7L2.

TCF7L2 is part of the GSK3/β-Catenin signaling pathway that regulates metabolism and a single SNP in the *TCF7L2* gene is, to date, the strongest genetic marker associated with T2D^28^. While *TCF7L2* was shown to be hypermethylated in adipose tissue upon a six month exercise training invention^27^, we did not find a significant association between lifelong exercise training and promoter methylation of TCF7L2 in muscle tissue. However, the finding, that the protein level of the upstream regulator, GSK3β, was significantly higher in the group of physically active than in the inactive men, supports a role of insulin- and/or Wnt-mediated GSK3 signaling in harnessing of the promoter potential of metabolic genes (Fig. 3A,B). Besides the insulin- and Wnt-GSK3 regulated genes, promoters of genes controlled by MYC, GATA3 and the polycomb complex were also found to be hypomethylated. These transcription factors might thereby also contribute to exploitation of the larger transcriptional potential in the trained group.

While genes encoding components of the mitochondrial respiratory complexes were not significantly hypomethylated, the protein levels of Complex I–IV were significantly higher in the physically active than in the inactive group in agreement with previous studies^29,30^. This regulation might be associated with the observed hypomethylation of the *TFAM* promoter, because TFAM is responsible for stability of mtDNA and transcriptional regulation of mitochondrial genes encoding subunits in the mitochondrial complexes^14^.

Notably, although the total bodyweight of the two subject groups were similar, the physically active men had a higher muscle mass, and correspondingly lower fat mass than the inactive (Table 1). This suggests that the observed epigenetic regulation of myogenic factors in skeletal muscle by lifelong physical activity (Fig. 4) may contribute to maintaining muscle mass during aging. This is supported by the current findings that principal components of the CDC42 pathway displayed promoter hypomethylation in the active compared with the inactive group. The CDC42 pathway is involved in regulation of actin/myosin cross-linking and actin polymerization^19^, suggesting that promoter hypomethylation of genes encoding components in the CDC42 signaling pathway is also involved in the preservation of muscle contractile function during aging. Furthermore, the observation that the promoter of *MYOD1* was hypomethylated in active relative to inactive could indicate that the MyoD transcription factor contributes to maintenance of muscle mass. This is in accordance with other findings, where muscle cells retain hypermethylated *MYOD1* when encountering a high inflammatory stress in early stages of their proliferatory life. These muscle cells were also more susceptible to muscle wasting parameters at later stages of life versus controls when encountering stress^31^. Our data are also in accordance with a 3-month human exercise training study that revealed hypomethylation of genes involved in myogenesis, muscle structure, function and bioenergetics^32^. Thus, we propose that epigenetic regulation of genes encoding myogenic and structural/contractile proteins may be part of an epigenetic memory that allows for a higher regenerative potential of skeletal muscle after exposure to lifelong physical activity.

However, the molecular mechanisms behind the differences in DNA methylation level between the two groups of men were not immediately evident from the present results. Although the DNA-methyltransferase gene *DMNT3B* was found to be hypomethylated in the physically active group relative to the inactive, this was not associated with altered levels of DMNT3B protein, as would be expected if the hypomethylation of the promoter directly influenced *DNMT3B* expression. In addition, the lack of difference in DNMT3B protein level between the two groups does not support that differences in DNMT3B protein level caused the observed general hypomethylation in the physically active relative to the inactive group. Because the level of DNMT3B has recently been shown to be increased in the cortex of both young and old rats in response to exercise training^33^, it may be speculated that species or tissue differences play a role in exercise training-induced regulation of *DNMT3B*. In addition, difference in DNMT3B activity between active and inactive cannot be ruled out.

In conclusion, the present study provides a gene signature of 745 genes that are regulated by lifelong physical activity at the epigenetic level in skeletal muscle from aged men. The results suggest that lifelong physical activity leads to epigenetic modifications in skeletal muscle influencing expression of genes with potential impact on insulin sensitivity, glycolytic and oxidative metabolism and muscle regeneration. Our study does not include young subjects and we can therefore not determine whether lifelong physical activity prevents an age-associated change or is merely an exercise training-induced effect. However, we propose that lifelong physical activity promotes an epigenetic memory that serves to maintain skeletal muscle function during aging.

## Methods

### Healthy human subjects

Two groups of healthy male subjects, sedentary and physically active were recruited through newspaper advertisements. Members of the two groups were further selected based on a questionnaire addressing exercise habits throughout life, medication and known diseases. Subjects were included in the study if they were declared healthy and had no known metabolic or cardiovascular diseases. In addition, blood sample screening of lipid profile, inflammatory states and blood pressure further confirmed a healthy lifestyle. The sedentary group (n = 8, age 62.8 ± 1.3 years) had lived a lifelong sedentary lifestyle with physical activity at most one time per week throughout their life, while the physical active group (n = 8, age 62.1 ± 1.4 years) had exercised more than three times per week throughout their life. The reported physical activity differed over the years for several of the participants and included soccer, bicycling, hiking, running, gymnastics, handball, badminton, military training, fitness, swimming, tennis, boxing, track and field and ice hockey. Moreover, the lifelong physically active subjects trained for at least 5 hours/week at the time of the experiment. Alcohol and smoking are unlikely to be confounding factors, although three physically inactive subjects and one physically active subject were smokers, while all other subjects were nonsmokers, while the alcohol consumption varied more within groups than between groups. The diet of the subject was not recorded and diet can therefore not be ruled out as a potentially confounding factor. Ethical approval was granted by the Committee of Copenhagen and Frederiksberg communities, reference number H-7-2014-001 and the research was conducted in accordance with the guidelines of *The Declaration of Helsinki*. After being informed verbally and in writing of the experimental procedures and associated risks, all the participants gave their written consent to take part in the study.

Measurements of percent body fat by DEXA scanning confirmed the group differences (30.5 ± 7.9 and 21.9 ± 4.2 percent in the sedentary and active group, respectively). An incremental ergometer cycling challenge was performed consisting of cycling at 120 watts for 5 minutes, followed by 20 watt increments every second minute until reaching perceived exertion of 18 on the Borg scale, at which participants continued until exhaustion. The cycling test confirmed the difference in endurance exercise performance between groups, while no significant differences were documented for age, height, weight or blood levels of cholesterol, testosterone, inflammatory markers or leukocyte count (Table 1).

On the day of the experiment, subjects arrived at the laboratory late afternoon (5 PM) having fasted at least 2 hours prior to experiment. Under local anaesthesia, muscle biopsies from *vastus lateralis* were collected (Bergström needle), transferred into liquid nitrogen and stored longterm at −80 °C. The same group of men was also tested for presence of extrachromosomal circular DNA in muscle and lymphocytes which is described in^20^.

### Whole genome DNA methylation library preparation

The genome-wide mapping of 5-methylcytosine in DNA from muscle was carried out by whole genome bisulphite sequencing. For this purpose, 10–20 mg of muscle tissue was homogenized with the TissueRuptor (QIAGEN) in 360 µl ATL buffer (QIAGEN) on ice. DNA was subsequently extracted with the DNeasy Tissue Mini Kit according to protocol (QIAGEN) treating homogenized tissue sample with proteinase K for 6 hours. The bisulphite conversion was performed on 500 ng input DNA using EZ DNA Methylation Kit (Zymo). Promega unmethylated control Lambda DNA was spiked in the DNA samples to serve as a control for measuring bisulphite conversion rate. After bisulphite conversion, the library preparation was done by Illumina Epicenter methylSeq kit according to the manufacturer’s instructions. Subsequently each individual DNA library sequenced in three lanes of HiSeq2500 machine achieving approximately on average a base coverage more than 20X.

### Whole genome DNA methylation data processing

The quality of the reads were measured by FASTQC (version 0.10.1) program as implemented in the trime galore wrapper (http://www.bioinformatics.babraham.ac.uk). Low quality base calls were removed from the 3′ end of the reads before trimming the adapter sequences. Our cut off for base quality control was Phred quality score of 20. Subsequently, Cutadapt detected and trimmed the adapter sequences from the 3′ end of reads. BSMAP aligner^34^ was used for mapping reads against the human genome hg19. For post data analyses purposes, we used an R library package methylKit^35^ and custom scripts implemented in R and Python. We used the MethylKit package to calculate methylation percentages per each single CpG. Percent methylation values for CpG dinucleotides were calculated by dividing the number of methylated Cs by the total coverage on that base. CpGs with at least 8X read coverage and at least a Phred score threshold of 20 were retained for calling CpG methylation.

### DNA methylation level at single CpG sites and genomic regions

DNA methylation level was calculated for each sample both at single CpG sites and genomic regions. Genomic regions (hg19) include CpG islands, CpG shores (defined as 2000 bp flanking regions on each side of the CpG island), promoters (defined as −2000 bp, +1000 bp around the TSS), and gene body (introns and exons). For scoring DNA methylation in our analyses, each individual CpG was required to pass a minimum Phred base quality score of 20 and be covered by at least 8 reads. To score DNA methylation over a region, the region had to contain at least three CpGs each covered by at least 8 reads. In order to identify differentially methylated regions (DMRs) in gene promoters, each region was scored for being DMR using the Fisher’s exact test. The p-values were adjusted for multiple testing using the sliding linear model (SLIM) method that corrects p*-*values to q-values as implemented in the methylKit package^36^. Promoters that met or exceeded a q-value of 0.001 and at least 30% absolute methylation differences were reported as being differentially methylated. It is of note that if a CpG island overlaps a promoter region, it is counted once for the CpG island category and once for the promoter region category.

### PCA analyses of methylation data

For principal component analysis (PCA), methylation per CpG nucleotide was collected as a matrix format; each row represented samples, that is 16 subjects; eight for active men and eight for physically inactive men. Each column represented a CpG feature. Mean, standard deviation, p-value and q-value were computed for each CpG locus of the active group and inactive group. Methylated CpGs with q-value < 0.001 were selected to be used for PCA analysis. During feature preprocessing, missing data either in the active group or inactive group were separately imputed by a weighted mean of the k nearest-neighbor algorithm (k = 4). Given the matrix, mean and standard deviation were computed for each CpG locus of the group of physically active and inactive men. We further removed CpG nucleotides that were not covered in each group. CpG loci were next sorted by the mean differences between the groups. CpG loci that had over 0.15 methylation level difference and standard deviation less than 0.01 between the two groups were considered significantly altered. A total of 26 CpG loci were selected after feature preprocessing. The data are standardized to z-score and were plotted using “svd” algorithm as implemented in Matlab software.

### Pathway Analyses

We used Enrichr^37^ to examine enriched histone marks (Epigenomics Roadmap HM ChIP-seq) and transcription factor binding sites TFBS (ENCODE_TF_ChIP-seq_2015) in ENCODE human cell lines based on the program Enrichr. For histone marks enrichment analyses (Epigenomics Roadmap HM ChIP-seq), we used data only for muscle tissues. It is of note that we used all hypo-methylated and hyper-methylated DMRs as input for EnrichR program. Moreover, we performed TFBS enrichment analyses based on ENCODE human cell lines that may not necessarily reflect the behavior of TFBS in human muscle tissues.

### Protein quantification, carbonylation and enzyme activity

Muscle lysate was prepared from ~10 to 15 mg dry weight of muscle as previously described^38^. Supernatant was collected following centrifugation for 20 min at 16,000 g (4 °C) and total protein content in the lysates was measured using the bicinchoninic acid assay (Thermo Fisher Scientific). Target proteins were analyzed by SDS‐PAGE and western blotting with equal total amount of protein loaded in each well of the SDS-gels. Primary antibodies were used to detect OXPHOS subunits (Abcam 110413), SOD2 (Millipore 06–984), Catalase (SC 50508), ANT1 (Abcam 102032), ANT2 (Abcam 118125), GSK3beta (Cell Signaling 9315), PDH-E1alpha (kindly provided by Professor Grahame Hardie, University of Dundee, Scotland). Membrane imaging and band quantification were performed using ImageQuant LAS 4000 (GE Healthcare, Little Chalfont, UK) and ImageQuant TL v8.1 software (GE Healthcare). Citrate synthase activity was measured with acetyl-CoA and oxaloacetate as substrate^39^ and protein carbonylation was measured with the OxiSelect^TM^ ELISA-kit (Cell Biolabs).

### Statistics

Values are presented as means ± standard error. A Student paired *t* test was applied to test the effect of lifelong physical activity (untrained versus trained subjects) on basal *in vivo* and molecular parameters in the muscle biopsies. Significance was accepted at *P* < 0.05. The statistical tests were performed using Sigmaplot 13.0 (Systat, USA).

## Supplementary information

all

Dataset 1

Dataset 2

Dataset 3

Dataset 4

Dataset 5

## Data Availability

All relevant data are within the paper and its Supporting Information files. All the raw data are under submission to the EMBL-EBI European Genome-phenome Archive (http://www.ebi.ac.uk/ega/).
