# Supplementary material for: Lifelong physical activity is associated with promoter hypomethylation of genes involved in metabolism, myogenesis, contractile properties and oxidative stress resistance in aged human skeletal muscle
Source: Sci Rep. 2019 Mar 1;9:3272. doi: 10.1038/s41598-018-37895-8 (PMC6397284; doi:10.1038/s41598-018-37895-8)
Supplement: Supplementary file 1 — all [file 41598_2018_37895_MOESM1_ESM.pdf]

Lifelong physical activity is associated with promoter hypomethylation of genes involved in metabolism, myogenesis, contractile properties and oxidative stress resistance in aged human skeletal muscle

M. Reza Sailani[1#], Jens Frey Halling[2#], Henrik Devitt Møller[2], Hayan Lee[1], Peter Plomgaard[3], Henriette Pilegaard[2], Michael P. Snyder[1§], Birgitte Regenberg[2§*]

[1] Department of Genetics, Stanford University School of Medicine, Stanford, USA.

[2] Department of Biology, University of Copenhagen, Denmark.

[3] The Centre of Inflammation and Metabolism, The Centre for Physical Activity Research, Department of Clinical Biochemistry, Rigshospitalet, Copenhagen University Hospital, Denmark.

Supplementary data

chr	start	end	strand	p-value	q-value	meth-diff
chr1	241798232	241801232	-	0	0	37,8514767
chr1	241798232	241801232	-	0	0	37,8514767
chr1	192542856	192545856	+	0	0	36,60778743
chr1	51886793	51889793	-	0	0	33,56848515
chr1	235488664	235491664	+	0	0	33,14708146
chr1	75041113	75044113	+	0	0	27,3394546
chr1	197626499	197629499	-	0	0	27,18631732
chr1	21945543	21948543	-	0	0	26,18707255
chr1	178837445	178840445	-	0	0	25,87166837
chr1	223987512	223990512	-	0	0	25,4831555
chr1	213185760	213188760	-	0	0	25,34230779
chr1	234763056	234766056	+	0	0	23,88391789
chr1	193149692	193152692	-	0	0	22,79168874
chr1	169335193	169338193	+	0	0	21,79450788
chr1	169335193	169338193	+	0	0	21,79450788
chr1	152195672	152198672	-	0	0	21,52759524
chr1	152195672	152198672	-	0	0	21,52759524
chr1	28158911	28161911	+	0	0	21,31421972
chr1	62316170	62319170	+	0	0	20,85959321
chr1	150668672	150671672	-	0	0	20,55069849
chr1	150668672	150671672	-	0	0	20,55069849
chr1	147372945	147375945	+	0	0	20,54113768
chr1	149367293	149370293	+	0	0	20,53000408
chr1	149367293	149370293	+	0	0	20,53000408
chr1	35645083	35648083	-	0	0	19,61618962
chr1	19228293	19231293	-	0	0	19,61530973
chr1	60236466	60239466	+	0	0	19,58617798
chr1	60236466	60239466	+	0	0	19,58617798
chr1	84377036	84380036	+	0	0	19,51608449
chr1	152481319	152484319	+	0	0	19,25522023
chr1	67134677	67137677	+	0	0	19,18350447
chr1	158799167	158802167	+	0	0	19,14779679
chr1	145411190	145414190	+	0	0	19,03318938
chr1	216823011	216826011	+	0	0	18,77482286
chr1	154530120	154533120	-	0	0	18,6563757
chr1	170488887	170491887	-	0	0	18,64647303
chr1	45986610	45989610	-	0	0	18,61719384
chr1	144289006	144292006	-	0	0	18,53526315
chr1	205743610	205746610	-	0	0	18,5052149
chr1	152645790	152648790	+	0	0	18,44537828

Supplementary Dataset 1.

List and chromosomal loci of all genes carrying methylated promoter regions in two groups of aged men having either engaged in lifelong physical activity or led a sedentary lifestyle. Absolute methylation difference between active and inactive human subjects is indicated (meth-diff).

chr	start	end	strand	p-value	q-value	meth-diff
chr7	48315690	48318690	+	0	0	30,3968768
chrX	74375175	74378175	-	0	0	45,6616519
chr17	48754259	48757259	+	0	0	31,770303
chr16	72697067	72700067	+	0	0	33,9480617
chr2	79410356	79413356	+	0	0	30,2300759
chr15	93441550	93444550	+	0	0	53,8170583
chr17	25948560	25951560	+	0	0	31,1084171
chr16	20497023	20500023	+	0	0	38,433569
chr17	35478716	35481716	-	0	0	34,7555458
chr16	20779305	20782305	+	0	0	39,3923745
chr14	71000732	71003732	-	0	0	42,4795481
chr8	24149579	24152579	+	0	0	38,7501322
chr8	24239797	24242797	+	0	0	35,3181153
chr21	28324279	28327279	+	0	0	34,1343197
chr15	73066502	73069502	-	0	0	42,8031816
chrX	49769257	49772257	+	0	0	31,5765525
chr10	88751118	88754118	+	0	0	30,3951784
chrX	115299957	115302957	+	0	0	38,8321357
chr7	137759177	137762177	+	0	0	33,0239181
chr21	29599986	29602986	-	0	0	34,3494238
chr14	101400827	101403827	+	0	0	48,7083445
chr14	20501917	20504917	-	0	0	37,7910799
chrX	109560380	109563380	-	0	0	31,7208693
chr1	104116062	104119062	+	0	0	35,790108
chr17	54303682	54306682	+	0	0	35,3602831
chr22	17155430	17158430	-	0	0	34,8344604
chr21	23468935	23471935	+	0	0	33,6392369
chrX	15872137	15875137	-	0	0	49,1936474
chrX	55023967	55026967	-	0	0	38,6879406
chrX	84256897	84259897	+	0	0	34,8560307
chr9	32996346	32999346	-	0	0	36,716671
chrX	66786682	66789682	+	0	0	33,2482745
chr20	47536274	47539274	+	0	0	30,385988
chr16	24967857	24970857	-	0	0	38,5606296
chr10	24910763	24913763	-	0	0	33,5209152

Supplementary Dataset 2.

List and chromosomal loci of genes displaying hypomethylated promoter region in physically active relative to inactive aged human subjects. Promoters were regarded as differentially methylated when reaching at least 30% absolute methylation difference and a false discovery rate (q-value) of less than 0.001.

chr	start	end	strand	p-value	q-value	meth-diff	Gene-Name
chr12	22687278	22690278	-	0	0	-53,696589	C2CD5
chr6	45164507	45167507	-	0	0	-41,580249	MIR586
chr6	45164507	45167507	-	0	0	-41,580249	SUPT3H
chrY	6741068	6744068	-	3,34E-14	1,51E-15	-38,968817	AMELY
chr10	59063319	59066319	-	0	0	-37,36958	MIR3924
chrM	14888	17888	-	0	0	-37,33982	MT-CYB
chrM	14888	17888	-	0	0	-37,33982	MT-TP
chrM	14888	17888	-	0	0	-37,33982	MT-TT
chr3	167190920	167193920	-	0	0	-36,437011	SERPINI2
chr11	27074846	27077846	+	0	0	-36,374625	BBOX1
chr11	27074846	27077846	+	0	0	-36,374625	RP11-1L12
chr13	38356497	38359497	-	0	0	-36,127253	TRPC4
chr14	62451802	62454802	+	0	0	-34,496419	SYT16
chr9	20714103	20717103	+	0	0	-34,447737	FOCAD
chr9	20714103	20717103	+	0	0	-34,447737	MIR491
chr14	57698090	57701090	-	0	0	-33,842742	EXOC5
chr4	66929099	66932099	+	0	0	-33,561821	RP11-25H12
chr3	168850758	168853758	-	0	0	-33,201269	MECOM
chr7	123239920	123242920	+	0	0	-32,988193	ASB15
chr7	123239920	123242920	+	0	0	-32,988193	RP11-390E23
chr12	10559388	10562388	-	0	0	-32,637456	KLRC4
chr8	79501465	79504465	+	0	0	-32,227566	PKIA
chr6	131892343	131895343	+	0	0	-31,824114	ARG1
chr6	131892343	131895343	+	0	0	-31,824114	MED23
chr11	18257384	18260384	-	0	0	-31,083779	SAA2-SAA4
chr11	18257384	18260384	-	0	0	-31,083779	SAA4
chr12	55862922	55865922	-	0	0	-31,028829	OR6C70
chr12	55862922	55865922	-	0	0	-31,028829	RP11-110A12

Supplementary Dataset 3.

List and chromosomal loci of genes displaying hypermethylated promoter region in physically active relative to inactive aged human subjects. Promoters were regarded as differentially methylated when reaching at least 30% absolute methylation difference and a false discovery rate (q-value) of less than 0.001.

Term	Overlap	P-value	Adjusted P-v	Old P-value	Old Adjusted	Z-score	Combined Sc	Genes
TCF7L2_HEK293_hg19	73/2000	0,57329524	1	2,17727E-05	0,01767942	-1,6431251	0,91415991	OTUD5;KDM
GATA3_SH-SY5Y_hg19	69/2000	0,75518036	1	0,000190455	0,06252232	-1,5967951	0,44837795	MTMR10;CS
POLR2AphosphoS5_SK-N-MC_	45/1154	0,38202897	1	0,000230994	0,06252232	-1,7756389	1,70862426	ERCC6L;CSRN
E2F4_HeLa-S3_hg19	42/1173	0,61501094	1	0,001832892	0,25570461	-1,6880656	0,82059438	PRPS1;ERCC6
CHD1_MEL cell line_mm9	28/691	0,33944481	1	0,002029455	0,25570461	-1,831276	1,97859105	RPL10;MRPS
EZH2_K562_hg19	64/2000	0,90915327	1	0,002050007	0,25570461	-1,6260642	0,15486894	MRPS11;CXO
STAT3_MCF 10A_hg19	108/3788	0,99938104	1	0,003028351	0,25570461	-0,566293	0,00035062	PCGEM1;OT
MYC_MCF 10A_hg19	98/3382	0,99783949	1	0,003043948	0,25570461	-0,9113038	0,00197101	ACSM3;ZMY
POLR2AphosphoS5_GM12878	48/1431	0,78662891	1	0,003138101	0,25570461	-1,6854775	0,40451235	ZNF493;ABC
POLR2AphosphoS2_MEL cell li	63/2000	0,9287039	1	0,003149072	0,25570461	-1,5112797	0,11178229	OTUD5;FMR
IKZF1_GM12878_hg19	62/2000	0,94491737	1	0,004762377	0,28638768	-1,5524169	0,08795652	SMG1;CHD9;
CEBPB_IMR-90_hg19	62/2000	0,94491737	1	0,004762377	0,28638768	-1,5478634	0,08769853	OTUD5;KDM
POLR2A_heart_mm9	62/2000	0,94491737	1	0,004762377	0,28638768	-1,483853	0,08407184	CHRM2;SPIN
ZMIZ1_MEL cell line_mm9	46/1392	0,81322595	1	0,004937719	0,28638768	-1,6458416	0,34027165	FOCAD;ADPG
EZH2_HeLa-S3_hg19	61/2000	0,95812284	1	0,007089672	0,31982299	-1,5469124	0,06617581	KDM5C;CHIC
KDM5A_H1-hESC_hg19	61/2000	0,95812284	1	0,007089672	0,31982299	-1,4958905	0,06399313	ZNF493;CXO
SIRT6_K562_hg19	61/2000	0,95812284	1	0,007089672	0,31982299	-1,4930369	0,06387105	ADPGK;BRCA
E2F4_MCF 10A_hg19	61/2000	0,95812284	1	0,007089672	0,31982299	-1,4904135	0,06375882	KDM5C;CHM
MYC_H1-hESC_hg19	84/2971	0,99804821	1	0,01103697	0,47168523	-0,9136502	0,00178499	OTUD5;KDM
POLR2AphosphoS5_neural cell	52/1709	0,94646821	1	0,013013803	0,5283604	-1,5706129	0,08641181	CSRNP3;MRP
TBP_GM12878_hg19	59/2000	0,97697315	1	0,014980127	0,52886362	-1,4173865	0,03301959	KDM5C;ZNF4
EZH2_mammary epithelial cell	59/2000	0,97697315	1	0,014980127	0,52886362	-1,3732056	0,03199035	KRTAP24-1;P
ZNF143_GM12878_hg19	59/2000	0,97697315	1	0,014980127	0,52886362	-1,3597478	0,03167684	SMG1;ZNF49
MXI1_HepG2_hg19	58/2000	0,98335912	1	0,021257446	0,69611782	-1,3326228	0,02236261	MTMR10;ER
GABPA_MCF-7_hg19	59/2048	0,98636527	1	0,02271221	0,69611782	-1,3991317	0,01920803	KDM5C;ZNF4
EP300_HeLa-S3_hg19	52/1770	0,97139834	1	0,023121685	0,69611782	-1,4842265	0,04307026	CRISP3;MRPS
POLR2A_MCF 10A_hg19	86/3155	0,99958337	1	0,023146775	0,69611782	-0,5442581	0,0002268	OTUD5;MTM

Supplementary Dataset 4.

List of pathways of enriched transcription factor binding sites based on the differentially methylated gene promoters in active relative to inactive subjects. Pathways were identified using Enrichr software with ENCODE human cell lines (https://www.encodeproject.org/) as reference.

Term	Overlap	P-value	Adjusted P-va	Z-score	Combined Score	Genes
H3K9ac Skele	38/508	0,0145636	0,08738161	-1,2252264	5,181763987	WFDC9;PRPS1;ERCC6L;CXORF21;MTMR8;ZCCHC18;LPA
H3K9me3 Ske	2/108	0,98323674	1	-0,1640001	0,00277248	OR4A47;FSCB
H3K36me3 Sl	3/174	0,98943167	1	-0,2603906	0,002766538	DNAJB7;CHML;SDHD
H3K27me3 Sl	26/1093	0,99999821	1	0,03741042	-6,70401E-08	CHRM2;WFDC9;BEX2;SAA4;HTR2C;COCH;BRS3;ADAMT
H3K4me3 Ske	224/8541	1	1	0,79593081	-8,72178E-11	CHIC1;ACSM3;UXT;RPS6KA3;GPR173;RPS6KA6;OPHN1
H3K4me1 Ske	85/4154	1	1	1,92766327	-4,7862E-11	ADPGK;MRPS11;ZNF45;DACH2;ELK1;MED12;MECOM;(

Supplementary Dataset 5.

List of enriched chromatin marks based on the differentially methylated gene promoters in active relative to inactive subjects. Chromatin marks were identified using Enrichr software with Roadmap project data as reference.

PDH E1a:

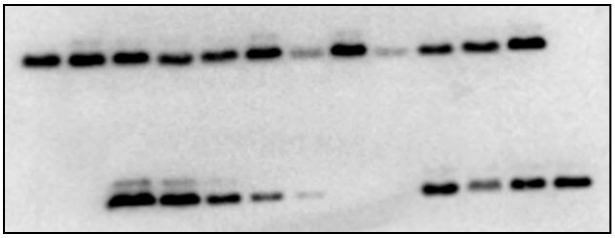

ANT1:

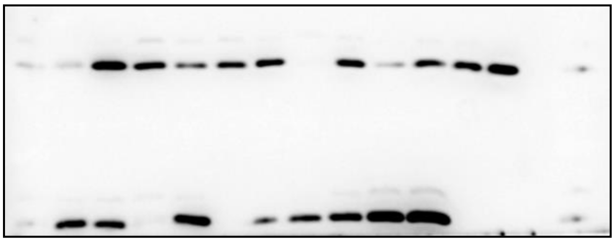

ANT2:

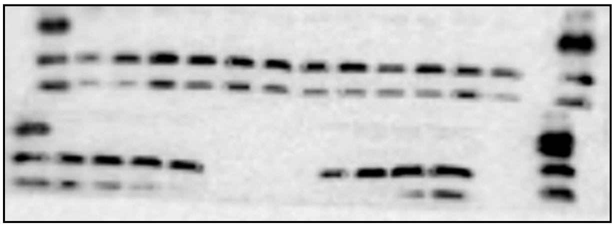

Catalase:

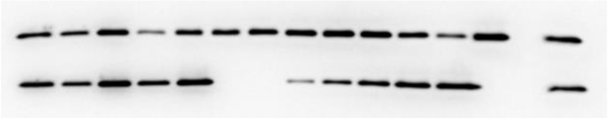

OXPHOS:

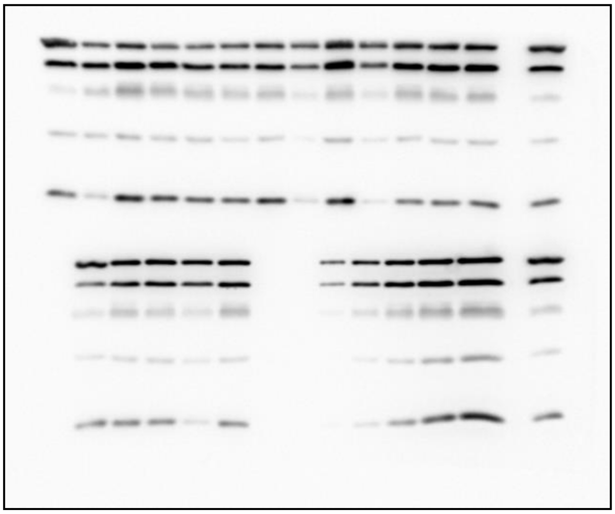

GSK3b:

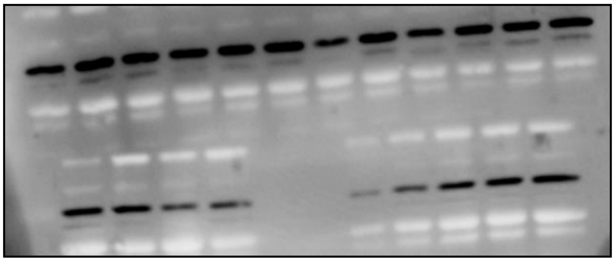

SOD2:

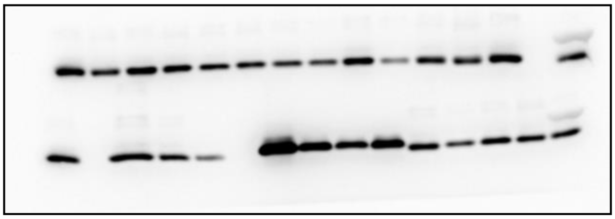

Supplementary Figure 1.

Full-length western blots for all presented proteins.

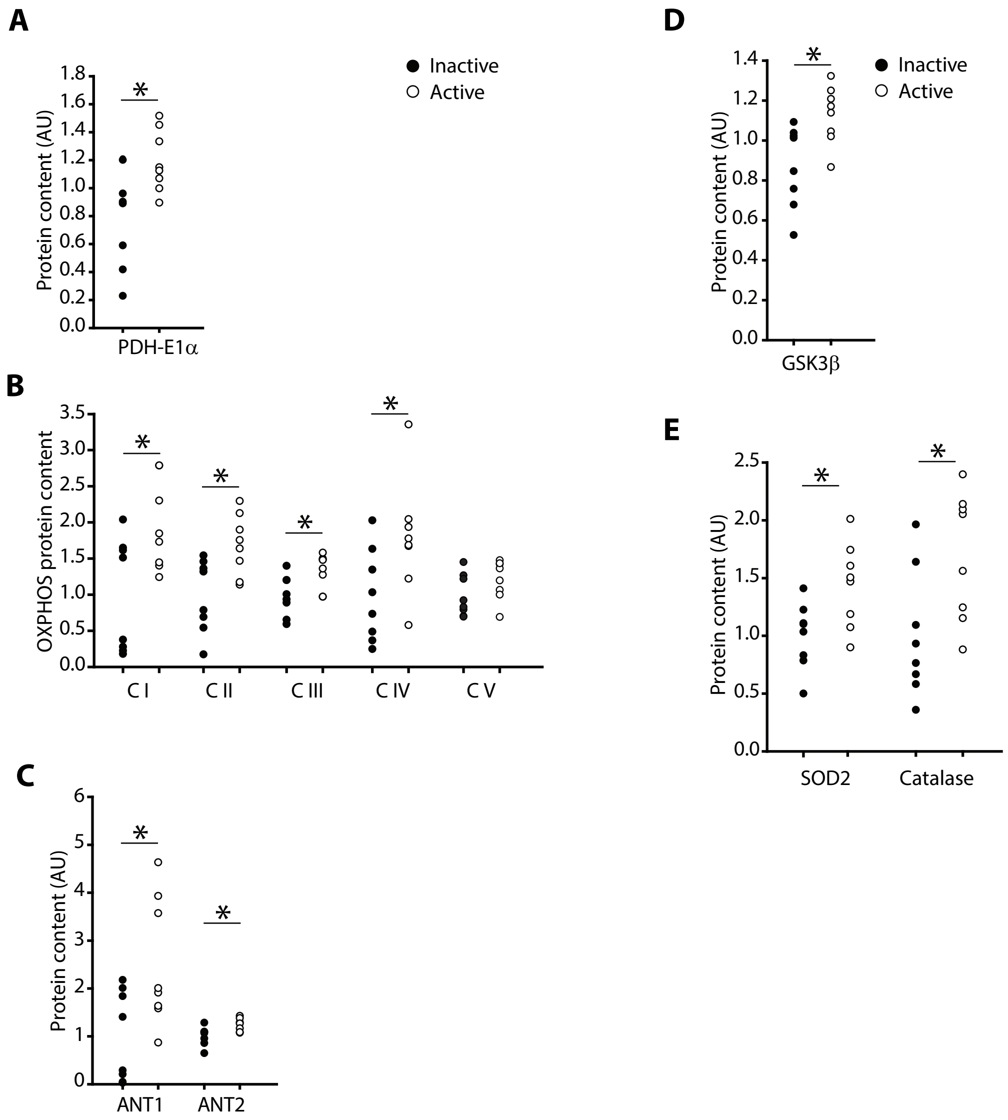

Supplementary Figure 2.

Scatter plots showing individual data points for all presented proteins.
